# Artificial intelligence to detect malignant eyelid tumors from photographic images

**DOI:** 10.1038/s41746-022-00571-3

**Published:** 2022-03-02

**Authors:** Zhongwen Li, Wei Qiang, Hongyun Chen, Mengjie Pei, Xiaomei Yu, Layi Wang, Zhen Li, Weiwei Xie, Xuefang Wu, Jiewei Jiang, Guohai Wu

**Affiliations:** 1 Ningbo Eye Hospital, Wenzhou Medical University, Ningbo, 315000 China; 2 School of Ophthalmology and Optometry and Eye Hospital, Wenzhou Medical University, Wenzhou, 325027 China; 3 Zunyi First People’s Hospital, Zunyi Medical University, Zunyi, 563000 China; 4 School of Computer Science and Technology, Xi’an University of Posts and Telecommunications, Xi’an, 710121 China; 5 Guizhou Provincial People’s Hospital, Guizhou University, Guizhou, 550002 China; 6 School of Electronic Engineering, Xi’an University of Posts and Telecommunications, Xi’an, 710121 China

**Keywords:** Translational research, Eyelid diseases

## Abstract

Malignant eyelid tumors can invade adjacent structures and pose a threat to vision and even life. Early identification of malignant eyelid tumors is crucial to avoiding substantial morbidity and mortality. However, differentiating malignant eyelid tumors from benign ones can be challenging for primary care physicians and even some ophthalmologists. Here, based on 1,417 photographic images from 851 patients across three hospitals, we developed an artificial intelligence system using a faster region-based convolutional neural network and deep learning classification networks to automatically locate eyelid tumors and then distinguish between malignant and benign eyelid tumors. The system performed well in both internal and external test sets (AUCs ranged from 0.899 to 0.955). The performance of the system is comparable to that of a senior ophthalmologist, indicating that this system has the potential to be used at the screening stage for promoting the early detection and treatment of malignant eyelid tumors.

## Introduction

Eyelid tumors are the most common neoplasm encountered in daily ophthalmology practice^1,2^. As eyelids have many tissue types, various benign and malignant tumors can develop^3^. Malignant eyelid tumors pose a great threat because of their proximity to the eyeballs, brain, and paranasal sinuses, which may cause cosmetic disfigurement and severe morbidity^4,5^. Early recognition and treatment of malignant eyelid tumors can result in the most cosmetically and functionally satisfactory outcomes^4–6^. In addition, although melanoma and sebaceous gland carcinoma (SGC) of the eyelid are rare lesions, they have high mortality^7,8^. However, the estimated 5-year survival rate of these malignant eyelid tumors can be over 99% if they could be detected in their earliest stages (depth of skin invasion ≤0.76 mm)^8^. Therefore, early detection of these malignant eyelid tumors is considerably critical.

Benign and malignant eyelid tumors sometimes have overlapping features, hence differentiation between them can be challenging for primary care physicians, dermatologists, and ophthalmologists without sufficient experience^4,5^. Due to the intricate anatomy of the eyelid, the diagnosis of eyelid tumors often requires experienced ophthalmologists. However, while over 200,000 ophthalmologists worldwide, there is a present and expected future shortfall in the number of ophthalmologists in both developing and developed countries^9^. The shortage of experienced ophthalmologists may hinder the early detection of malignant eyelid tumors, especially in underdeveloped countries and remote regions.

Recently, artificial intelligence (AI) has been reported to attain a high level of accuracy in the automated detection of numerous diseases from clinical images^10–15^. In ophthalmology, a large number of studies developed deep learning-based systems that could accurately detect ocular diseases such as diabetic retinopathy, retinal detachment, and glaucoma^16–21^. However, eyelid tumors, particularly malignant ones, which need early detection and prompt referral, are not well investigated. Employment of a deep learning algorithm in conjunction with eyelid tumor images may realize the early identification of malignant eyelid tumors with potential benefits including increased accessibility and affordability for the suspected cases. In addition, for allowing medical practitioners and suspected patients to proactively track eyelid tumors and identify malignant ones earlier, the algorithm should be capable of localizing eyelid tumors autonomously within images.

In this study, we tried to develop an AI system, which used a faster region-based convolutional neural network (Faster R-CNN) and deep learning classification networks, to automatically locate eyelid tumors and distinguish malignant from benign tumors in photographic images captured by ordinary digital cameras. Besides, we separately investigated the performance (dichotomous diagnosis: malignant, benign) of this system in detecting the most frequent malignant and benign eyelid tumors. Moreover, we compared the performance of the system to that of ophthalmologists of different levels.

## Results

### Dataset characteristics

After excluding 150 photographic images without histopathological diagnoses, a total of 1,417 images with 1,533 eyelid tumors delineated by tight bounding boxes were used to establish and evaluate an eyelid tumor detection system (ETDS). A total of 1,533 cropped images (1,161 images of benign tumors and 372 images of malignant tumors) created by the ETDS were leveraged to develop and assess the deep learning classification system. Details on the development set and external test set are described in Table 1.

**Table 1:** Characteristics of the development set and the external test set.

		Development set		External test set
Total no. of photographic images		1,258		309
Total no. of qualified photographic images^a^		1,151		266
Total no. of cropped images		1, 248		285
No. of patients		675		176
Mean age/range (years)		49.6 (1–100)		50.0 (2–87)
No. of women (%)		421 (62.4)		111 (63.1)
Institution		NEH		JEH and ZFPH
Location of institution		East of China		East and west of China
Camera model		Canon IXUS-130, Nikon COOLPIX-S7000		FUJIFILM FinePix-F41, HUAWEI EVA-AL00
	Training set	Validation set	Internal test set	
Benign eyelid tumor^b^	668/883 (75.7)	121/168 (72.0)	146/197 (74.1)	226/285 (79.3)
Malignant eyelid tumor^b^	215/883 (24.3)	47/168 (28.0)	51/197 (25.9)	59/285 (20.7)

*NEH* Ningbo Eye Hospital, *JEH* Jiangdong Eye Hospital, *ZFPH* Zunyi First People’s Hospital.

^a^Qualified photographic images indicate the images with unequivocal histopathological diagnoses.

^b^Data are no. of cropped images/total no. (%) unless otherwise indicated.

The top three malignant eyelid tumors in our datasets are basal cell carcinoma (BCC) (245/372, 65.9%), squamous cell carcinoma (SCC) (50/372, 13.4%), and SGC (66/372, 17.7%). The proportion of the other malignant eyelid tumors (e.g., melanoma and actinic keratosis) is 3.0% (11/372). The top three benign eyelid tumors are squamous cell papilloma (SCP) (104/1161, 9.0%), nevus (385/1,161, 33.2%), and seborrheic keratosis (106/1161, 9.1%). The proportion of the other benign eyelid tumors (e.g., hemangioma and xanthoma) is 47.4% (566/1161). In total, 116 (7.6%) malignant tumors and 630 (41.1%) benign tumors appeared on the upper eyelid, 238 (15.5%) malignant tumors and 470 (30.7%) benign tumors appeared on the lower eyelid, 11 (0.7%) malignant tumors and 55 (3.6%) benign tumors appeared in the inner canthus, and 7 (0.5%) malignant tumors and 6 (0.4%) benign tumors appeared in the outer canthus.

### Performance of the ETDS and different deep learning algorithms

**Fig. 1:**
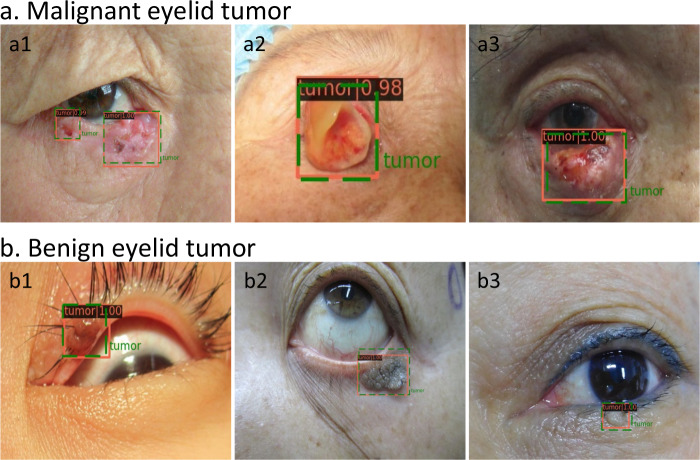
Representative detection results of the Faster-RCNN for eyelid tumors. The green dotted line boxes refer to the ground truth of the eyelid tumors. The orange solid line boxes refer to the detection results by Faster region-based convolutional neural network (R-CNN). The orange numerical values indicate confidence scores (range 0–1) which reflect how confident the model is that the box contains an eyelid tumor. **a** Malignant eyelid tumor: **a1** basal cell carcinomas. **a2** squamous cell carcinomas. **a3** sebaceous gland carcinoma. **b** Benign eyelid tumor: **b1** nevus. **b2** seborrheic keratosis. **b3** squamous cell papilloma.

**Fig. 2:**
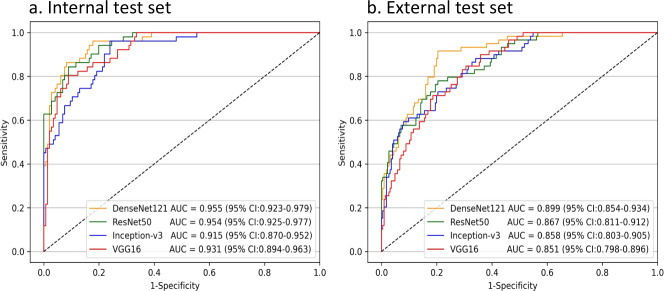
Performance of four deep learning algorithms in discerning malignant eyelid tumors. **a** The receiver operating characteristic (ROC) curves of the deep learning algorithms in the internal test set. **b** The ROC curves of the deep learning algorithms in the external test set. AUC area under the ROC curve. CI confidence interval.

The average precision (AP) scores of the ETDS for locating eyelid tumors were 0.801 in the internal test set and 0.762 in the external test set. The representative detection results of the Faster-RCNN for eyelid tumors were shown in Fig. 1. Four classic deep learning algorithms, DenseNet121, ResNet50, Inception-v3, and VGG16 were used to train models to distinguish malignant eyelid tumors from benign ones. The receiver operating characteristic (ROC) curves of these algorithms in internal and external test sets are shown in Fig. 2, and the corresponding confusion matrices are presented in Supplementary Fig. 1, which indicates that the optimal algorithm is the DenseNet121. The t-distributed stochastic neighbor embedding (t-SNE) technique also showed that the features of benign and malignant eyelid tumors learned by the DenseNet121 were more separable than those of the ResNet50, Inception-v3, and VGG16 (Supplementary Fig. 2).

In discerning malignant eyelid tumors, the optimal algorithm DenseNet121 achieved an area under the receiver operating characteristic curve (AUC) of 0.955 (95% confidence interval [CI], 0.923–0.979), a sensitivity of 96.1% (95% CI, 90.8–100), and a specificity of 77.4% (95% CI, 70.6–84.2) in the internal test set, and an AUC of 0.899 (95% CI, 0.854–0.934), a sensitivity of 91.5% (95% CI, 84.4–98.6), and a specificity of 79.2% (95% CI, 73.9–84.5) in the external test set. Further information encompassing accuracies, sensitivities, and specificities of these four algorithms is displayed in Table 2. Compared to the ground truth, the unweighted Cohen’s κ coefficients of the optimal algorithm DenseNet121 were 0.613 (95% CI, 0.497–0.730) in the internal test set and 0.560 (95% CI, 0.452–0.668) in the external test set.

**Table 2:** Performance of four deep learning algorithms in identifying malignant eyelid tumors in the internal and external test sets.

Deep learning algorithms	Internal test set			External test set		
	Sensitivity (95% CI)	Specificity (95% CI)	Accuracy (95% CI)	Sensitivity (95% CI)	Specificity (95% CI)	Accuracy (95% CI)
DenseNet121	96.1% (90.8–100)	77.4% (70.6–84.2)	82.2% (76.9–87.6)	91.5% (84.4–98.6)	79.2% (73.9–84.5)	81.8% (77.3–86.2)
ResNet50	94.1% (87.7–100)	77.4% (70.6–84.2)	81.7% (76.3–87.1)	69.5% (57.7–81.2)	84.5% (79.8–89.2)	81.4% (76.9–85.9)
Inception-v3	90.2% (82.0–98.4)	77.4% (70.6–84.2)	80.7% (75.2–86.2)	74.6% (63.5–85.7)	73.9% (68.2–79.6)	74.0% (68.9–79.1)
VGG16	86.3% (76.8–95.7)	78.8% (72.1–85.4)	80.7% (75.2–86.2)	66.1% (54.0–78.2)	82.3% (77.3–87.3)	78.9% (74.2–83.7)

CI, confidence interval.

**Fig. 3:**
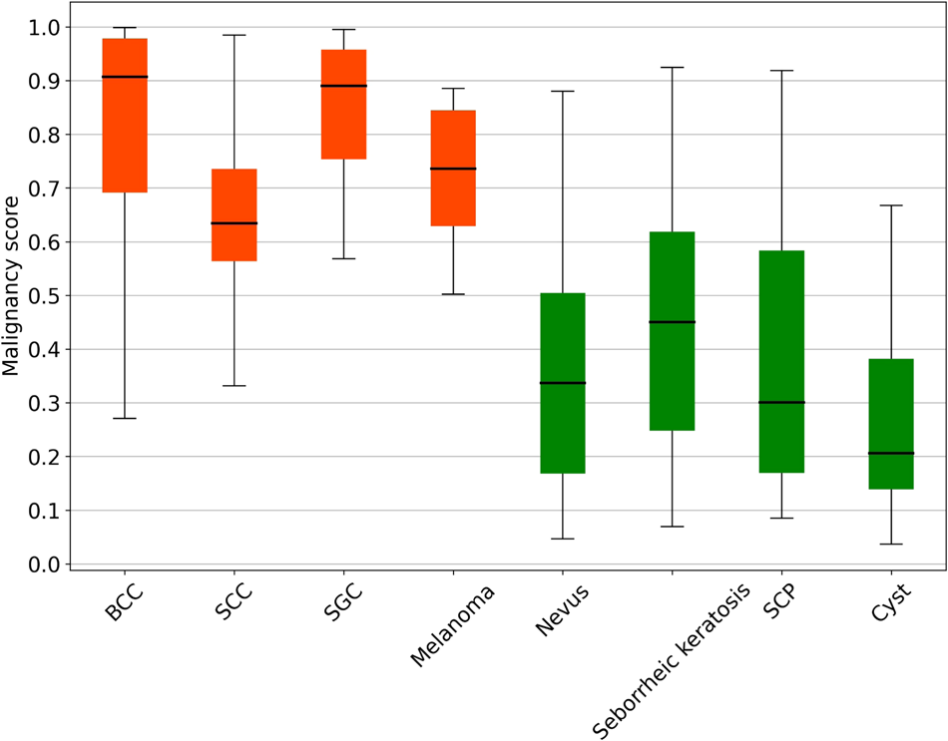
Malignancy scores (range 0–1) predicted by the deep learning classification system for the major categories of malignant and benign eyelid tumors. Scores closer to 1 denote a higher probability of malignancy. The upper and lower bounds of the box refer to the 25th and 75th percentiles, and the line intersection in the box refers to the median. Whiskers refer to the full range of malignancy scores. BCC basal cell carcinoma, SCC squamous cell carcinoma, SGC sebaceous gland carcinoma, SCP squamous cell papilloma.

The distribution of malignancy scores related to the major categories determined by the optimal algorithm DenseNet121 in internal and external test sets is shown in Fig. 3. With a malignancy cutoff at > 0.5, the percentage of correctly classified images in malignant tumors was 91.0% (61/67) in BCC, 92.9% (13/14) in SCC, 100% (19/19) in SGC, and 100% (7/7) in melanoma. In benign tumors, the percentage of correctly classified images was 70.2% (73/104) in nevus, 56.3% (18/32) in seborrheic keratosis, 69.2% (27/39) in SCP, and 90.0% (27/30) in cyst.

A total of 36 images (12 malignant tumor images and 24 benign tumor images) were classified into the borderline case group (eyelid tumors of uncertain malignant nature) by the expert. The optimal algorithm DenseNet121 achieved an accuracy of 77.8% (95% CI, 64.2–91.4) with a sensitivity of 83.3% (95% CI, 62.2–100) and a specificity of 75.0% (95% CI, 57.7–92.3) in differentiating malignant eyelid tumors from benign ones on this group. The receiver operating characteristic curve of our system in images of eyelid tumors of uncertain malignant nature is shown in Supplementary Fig. 3.

### Classification errors of the deep learning system

In total, 87 images (18.0% of the 482 images) from the internal and external test sets had inconsistent findings between the system and the ground truth. In the category of malignant eyelid tumors (110 images), seven images (6.4%) were misclassified by the system as benign tumors (false-negative classification). In the category of benign eyelid tumors (372 images), 80 images (21.5%) were misclassified by the system as malignant tumors (false-positive classification). The details regarding images misclassified by the system are illustrated in Supplementary Fig. 4. Examples of incorrectly classified images are shown in Supplementary Fig. 5.

**Fig. 4:**
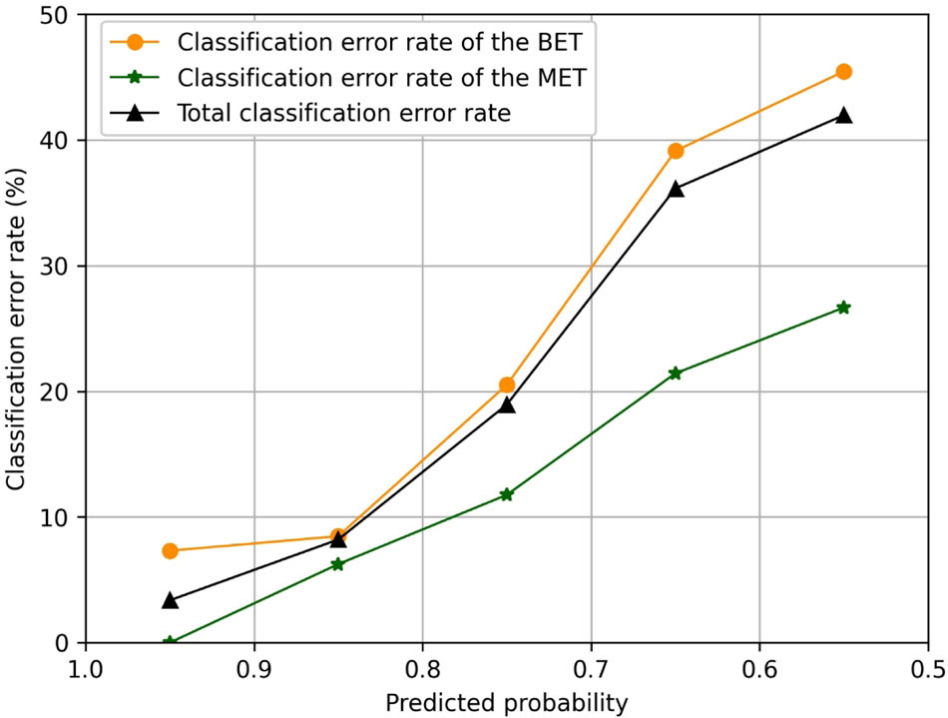
Relationship between the classification error rates and predicted probability values. The classification error rate is the fraction of incorrectly classified images in each predicted probability interval between the breaking points. BET benign eyelid tumor, MET malignant eyelid tumor.

The relationship between the classification error rates and predicted probabilities of our system is displayed in Fig. 4, which denoted that the classification error rate of each category and the total classification error rate increased with the decrease of the predicted probabilities. When the predicted probabilities are over 0.9, the classification error rate of the malignant eyelid tumors is 0% and the classification error rate of the benign eyelid tumors is about 8%. When the predicted probabilities are less than 0.7, the classification error rates of these two categories are both greater than 20% and the total classification error rate is over 35%. As our system is a binary classification system, the lowest predicted probability value of the system’s output is greater than 0.5.

### Interpretability of the deep learning system

**Fig. 5:**
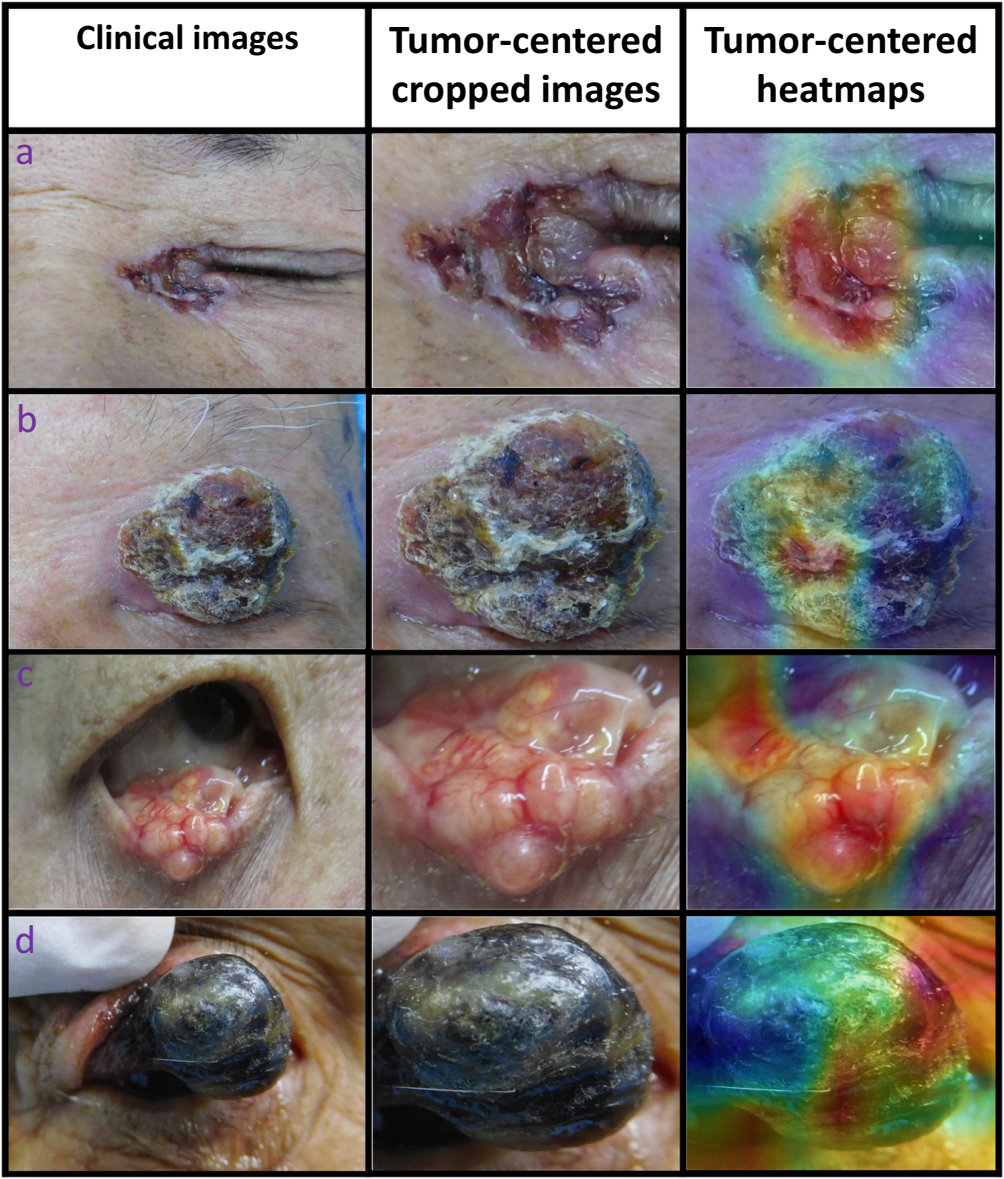
Examples of photographic images, cropped images and corresponding heatmaps of malignant eyelid tumors. **a** Basal cell carcinomas. **b** Squamous cell carcinomas. **c** Sebaceous gland carcinoma. **d** Melanoma.

**Fig. 6:**
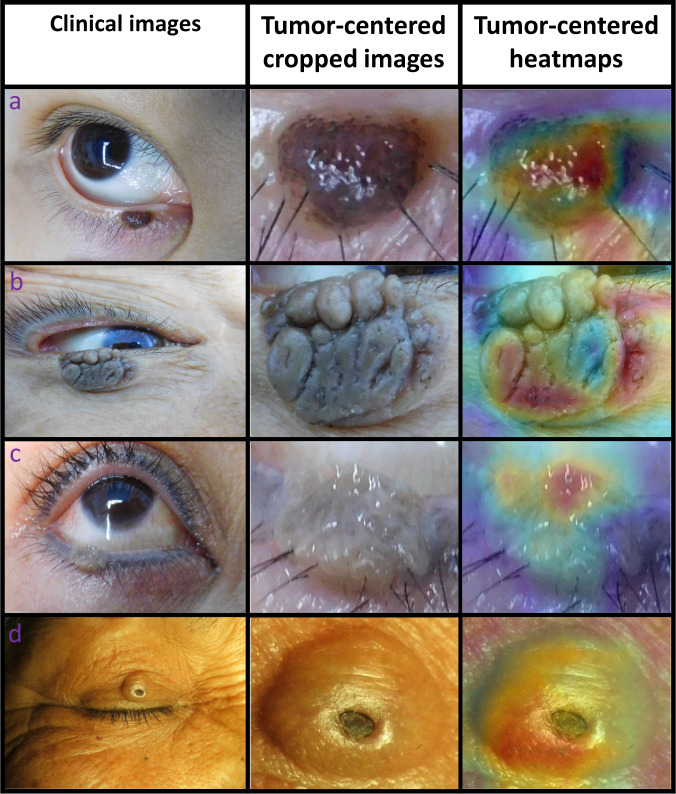
Examples of photographic images, cropped images and corresponding heatmaps of benign eyelid tumors. **a** Compound nevus. **b** Seborrheic keratosis. **c** Squamous cell papilloma. **d** Epidermoid cyst.

To investigate the interpretability of the system in classifying benign and malignant eyelid tumors, heatmaps were created to visualize the regions that contributed most to the system’s decisions. We found that heatmaps highlighted the regions of benign and malignant eyelid tumors, regardless of the size, location, and shape of the tumors. Examples (images and corresponding heatmaps) of malignant and benign eyelid tumors are shown in Figs. 5 and 6, respectively.

### Deep learning system versus ophthalmologists

For differentiating malignant eyelid tumors from benign ones based on the external test set, the junior ophthalmologist achieved an accuracy of 72.3% (95% CI, 67.1–77.5) with a sensitivity of 66.1% (95% CI, 54.0–78.2) and a specificity of 73.9% (95% CI, 68.2–79.6), the senior ophthalmologist achieved an accuracy of 77.9% (95% CI, 73.1–82.7) with a sensitivity of 74.6% (95% CI, 63.5–85.7) and a specificity of 78.8% (95% CI, 73.4–84.1), and the expert achieved an accuracy of 90.2% (95% CI, 86.7–93.6) with a sensitivity of 94.9% (95% CI, 89.3–100) and a specificity of 88.9% (95% CI, 84.8–93.0), while the system achieved an accuracy of 81.8% (95% CI, 77.3–86.2) with a sensitivity of 91.5% (95% CI, 84.4–98.6) and a specificity of 79.2% (95% CI, 73.9–84.5). The sensitivity of the system was superior to that of the junior and senior ophthalmologists and comparable to that of the expert, whereas the specificity of the system was only inferior to that of the expert (Supplementary Table 1). Confusion matrices of these three ophthalmologists are presented in Supplementary Fig. 6.

## Discussion

Our objective in this study was to evaluate the performance of a deep learning system in distinguishing malignant eyelid tumors from benign ones based on photographic images captured by ordinary digital cameras. The findings shown in Fig. 2 demonstrated that deep learning algorithms performed well in discerning malignant eyelid tumors and the algorithm DenseNet121 had better performance than the other three algorithms. The generalizability of our system was confirmed on the basis of its good performance (AUC 0.899, sensitivity 91.5%, specificity 79.2%) in the external test set, of which images were collected from two other hospitals. Besides, the agreement between the outputs of the system and the ground truth was substantial according to the unweighted Cohen’s κ coefficients, further verifying the reliability of our system. When compared to the ophthalmologists of different levels, the system’s sensitivity was higher than that of the junior and senior ophthalmologists and comparable to that of the expert, while the system’s specificity is lower than that of the expert. As a high sensitivity is a prerequisite in a potential screening tool^22^, the results implied that our system can potentially serve as an efficient approach for the early detection of malignant eyelid tumors, reducing the medical costs and workload via avoiding the need for the further examination of evidently benign eyelid tumors.

In both eastern and western countries, the top three malignant eyelid tumors are BCC, SCC, and SGC, and the top three benign eyelid tumors are SCP, seborrheic keratosis, and nevus^1,2,23–25^, which are consistent with the statistics of our datasets. The accuracy of our system in detecting these most frequent malignant tumors is greater than 90%. Although melanoma is a rare lesion on eyelids, it has considerable potential morbidity and mortality^4^. The early recognition and timely treatment of patients with melanoma are crucial for improving the prognosis^4,5^. Therefore, we investigated the performance of our system in images of melanoma. Inspiringly, the percentage of correctly classified images by the system in malignant eyelid tumors was 100% in melanoma. These results suggested that our system had good performance in identifying both frequent and rare malignant eyelid tumors.

Differentiating a malignant eyelid tumor from a benign one can be challenging for the examining physician in primary care centers due to the relatively small size, variability in clinical presentation, and minimal ophthalmologic training in the medical school^5,26^. Unlike skin tumors in other regions of the body, where physicians might feel comfortable conducting biopsies, the intricate anatomy of eyelids often calls for a referral to an ophthalmologist. Even oculoplastic ophthalmologists have only 70% accuracy in diagnosing eyelid tumors^5^. Due to the reliable performance, our system could be utilized both at the screening stage before patients visit the physician and at the disease confirmation stage after the consultation, promoting the early detection of malignant eyelid tumors.

Recently, Adamopoulos et al.^27^ trained models using a deep learning artificial neural network for classifying patients with eyelid BCC and healthy individuals without eyelid tumors based on 143 photographic images obtained from a single clinical center. The AUC of their best model reached approximately 1.00. As their model was mainly used for detecting eyelid BCC, it may not be employed to discern other malignant eyelid tumors. In comparison to their study, our study showed several important features. First, we developed a deep learning system that could distinguish a variety of malignant eyelid tumors from benign ones with AUCs ranging from 0.899 to 0.955. In addition, our system has the potential to be applied to ordinary digital cameras, which would be a convenient and cost-effective approach for promoting the early detection of malignant eyelid tumors. Third, our datasets included 1,417 photographic images collected from three different clinical hospitals and thereby were more representative of the data in real-world settings. Fourth, the ground-truth label of each image in this study is based on an unequivocal histopathological diagnosis.

While deep learning has great performance in medical image diagnosis problems, it remains highly criticized for being “a black box”^28^. This is considered as a major shortcoming in the application of deep learning to high-stakes decisions^29^. To explore this issue, we generated heatmaps using a Gradient-weighted Class Activation Mapping (Grad-CAM) technique to visualize the regions which contributed most to the system’s classification. The heatmap revealed that the eyelid tumors in images, irrespective of malignant and benign categories, were identified as the critical regions, which further demonstrated the validity of the system (Figs. 5 and 6). Our system’s interpretability feature could facilitate its application in real-world settings.

Compared to the ground truth, our system made a few mistakes. For the malignant eyelid tumors incorrectly classified as benign ones, 85.7% (6/7) images showed BCC. The BCCs in these images are relatively small, unclear, and similar to the scar and seborrheic keratosis, which might be possible contributors to this misclassification. For benign eyelid tumors misclassified as malignant ones, most images showed nevus (31/80, 38.8%), followed by seborrheic keratosis (14/80, 17.6%), and SCP (12/80, 15.0%). These benign tumors, in varying degrees, have similar appearances (e.g. irregular shapes, irregular pigmentation, and telangiectasia) to malignant ones. When analyzing the relationship between the system’s predicted probability and classification error rate, the results suggested that the higher classification error rate is associated with the lower predicted probability. Hence ophthalmologists need to pay more attention to the images with low predicted probability values. As an ideal intelligent screening system should minimize both false-negative and false-positive errors, further studies are needed to address this issue.

Although the present study proves the potential of the deep learning system in discriminating between malignant and benign eyelid tumors, the system has several limitations which we wish to address in the near future. First, since our system was developed solely based on the Chinese Population from several different geographic regions, its effectiveness in other racial populations would need to be further verified. Additional training on various clinical and demographic cohorts might further improve the performance and clinical utility of the system in a broad range of populations. In addition, while our system appears well suited for a screening purpose (discerning malignant eyelid tumors), it cannot provide a specific diagnosis based on images. We expect to collect more images of eyelid tumors of each category and then develop an AI system to realize this function.

In conclusion, the current study demonstrated that our deep learning system had roust performance in differentiating malignant eyelid tumors from benign ones. This AI system has the potential to assist medical practitioners and suspected patients to proactively track eyelid tumors and identify malignant ones earlier.

## Methods

### Image acquisition

For developing a deep learning system, a total of 1,258 photographic images (675 patients) were collected at NEH. The NEH dataset included subjects who presented for eye examinations and ophthalmology consultations due to the discovery of eyelid tumors. The images were captured between January 2010 and March 2021 using ordinary digital cameras. To better confirm the effectiveness and generalizability of the deep learning system, an additional dataset including 248 photographic images (129 patients) collected at Jiangdong Eye Hospital (JEH) and 61 photographic images (47 patients) collected at Zunyi First People’s Hospital (ZFPH) were used to externally assess the system. The images of the development set and the external test set were taken at various locations, such as outpatient clinics, inpatient wards, operating rooms, hence the lighting and background of the images were not uniform, indicating the richness and diversity of our datasets. All anonymized, unaltered images (size, 0.6–5.5 megabytes per image) were transferred to researchers for inclusion in the study. This study followed the recently published reporting guidelines for the clinical research involving AI: the CONSORT-AI extension^30^.

### Development of an eyelid tumor detection system

As one photographic image may show one or more eyelid tumors of different nature, this study first developed an ETDS using the Faster-RCNN, an object detection network depending on region proposal algorithms^31^, to automatically locate and crop eyelid tumors from photographic images. This step can also remove the background noise around tumors in photographic images for better training the subsequent deep learning-based classification networks. Each eyelid tumor in a photographic image was delineated by a tight bounding box for the training of the Faster-RCNN model. Each cropped image only contains one eyelid tumor. The pipeline of the ETDS is described in Supplementary Fig. 7.

### Ground truth and image classification

Two junior ophthalmologists who both had two-year clinical experience were recruited to annotate cropped images. The label of each cropped image was based on an unequivocal histopathological diagnosis which was considered as the ground truth of this study. Images without sufficient diagnostic certainty were excluded from the study. All images with clear diagnoses were classified by the study steering committee into two categories: malignant eyelid tumors (including premalignant ones) and benign eyelid tumors. The malignant eyelid tumors included BCC, SCC, SGC, etc. The benign eyelid tumors included SCP, seborrheic keratosis, nevus, etc.

### Development of a deep learning classification system

The cropped images created by the ETDS using the NEH dataset were randomly split at a 7:1.5:1.5 ratio for training, validation, and testing of a deep learning classification system. No overlap was allowed among training, validation, and internal test sets. For acquiring the best deep learning algorithm to identify malignant eyelid tumors, four state-of-the-art CNN architectures (DenseNet121, ResNet50, Inception-v3, and VGG16) were investigated in this study. The architectural characteristics of these four networks were described as follows:

1. DenseNet121: This network has 121 layers that are densely connected through jointing all preceding layers into subsequent layers to accomplish strengthened feature propagation and alleviate a vanishing-gradient issue^32^. Recently, DenseNet121 has been used to identify keratitis from slit-lamp images^20^.
2. ResNet50: This CNN is a 50-layer network that utilizes skip residual connections to bypass signals across layers, allowing for the increase in layers without compromising the ease of training^33^. ResNet50 has been employed to detect brain abnormality from fluorodeoxyglucose positron emission tomography images^34^.
3. Inception-v3: This network has 42 layers and consists of 10 inception modules which can decrease the number of parameters to be trained and thereby reduce the computational complexity^35^. Inception-v3 has been applied to identify age-related macular degeneration from fundus images^36^.
4. VGG16: This network contains 41 layers, of which, 16 layers have learnable weights^37^. It includes the features of the classical network’s simple structure while expanding the network’s depth via the flexible use of 3 × 3 convolution^37^. VGG16 has been used to detect breast cancer from histopathologic images^37^.

Transfer learning was adopted as it could promote the performance of the deep learning algorithms in the tasks of medical image classification^38^. Weights pre-trained for ImageNet classification (1,000 object classes) were leveraged to initialize the CNN architectures^39^. Image standardization was performed as a preprocessing step before training the models. Due to using the transfer learning approach, all cropped images were resized to 224 × 224 pixels for the DenseNet121, ResNet50, and VGG16 algorithms and to 299 × 299 pixels for the Inception-v3 algorithm. Image pixel values were normalized within the range of 0 to 1. Data augmentation techniques were adopted because they were capable of enhancing the robustness of CNN networks^40^. Random brightness, rotation, and horizontal and vertical flipping were applied to the images of the training set to augment the sample size to 6 times larger than the original size (from 883 to 5,298).

The PyTorch deep learning framework (version 1.6.0) was used to train, validate, and test our models. The DenseNet121, ResNet50, Inception-v3, and VGG16 were trained using 4 Nvidia 2080TI graphics processing units. The mini-batch size was set at 32 on each GPU to gain 128 images in one iteration. The average value of these samples was computed to update the trainable parameters. A variation of the stochastic gradient descent algorithm, adaptive moment estimation (ADAM) optimizer, was used with an initial learning rate at 0.001, β1 of 0.9, β2 of 0.999, and a weight decay of 1e-4. Each algorithm was trained for 80 epochs. During the training process, accuracy and cross-entropy loss were calculated on the training and validation sets after each epoch and utilized as a reference for model selection. Each time the accuracy increased or cross-entropy loss decreased, a checkpoint saved the model state and corresponding weight matrix. The model with the highest validation accuracy was selected for use on the internal test set.

The performance of the binary classification model was further evaluated on an independent external test set. The process of the development and assessment of the deep learning classification system is described in Supplementary Fig. 8. The t-SNE technique was applied to visualize the embedding features of each category learned by the system in a two-dimensional space^41^.

To investigate the performance of the system in eyelid tumors without evident malignant features (borderline cases), we recruited an expert with 15 years of clinical experience to read all images from the external test set and select images of eyelid tumors of uncertain malignant nature by evaluating the appearance of the tumors.

### Heatmap generation

The Grad-CAM technique^42^ was employed to produce visual explanations for the decisions from the system by superimposing a visualization layer at the end of the CNN model. This method leverages the gradients of any target concepts, flowing into the last layer of the CNN to generate a localization map highlighting the key regions in the image for predicting the concept^42^. Redder regions denote the more significant features of the system’s prediction. Using this tool, the heatmap was generated to interpret the rationale of the system on the discrimination between malignant and benign eyelid tumors.

### Analysis of misclassified images

In a post hoc analysis, a senior ophthalmologist who was not involved in the original analysis reviewed all false-negative and false-positive findings made by the system. To illustrate these discrepancies, the possible reasons for misclassified images were analyzed and documented on the basis of the observed characteristics in images. In addition, the relationship between the classification error rates and the system’s predicted probability was investigated.

### Performance comparison between the deep learning system and ophthalmologists

To evaluate our deep learning classification system in the context of malignant eyelid tumor detection, we recruited three ophthalmologists who had different levels of clinical experience (a junior ophthalmologist with three-year experience, a senior ophthalmologist with seven-year experience, and an expert with 15-year experience). The external test set was employed to compare the performance of the optimal system to that of ophthalmologists with the ground truth. Of note, to reflect the real level of the ophthalmologists in routine clinical practices, they were not informed that they competed with the system to avoid bias from the competition.

### Statistical analysis

The performance of the ETDS was evaluated by calculating the AP score using mmdetection 2.10.0. The sensitivity, specificity, accuracy, and AUC were calculated to assess the performance of the deep learning classification system. The 95% CIs of sensitivity, specificity, and accuracy were estimated with the Wilson Score approach utilizing a package of Statsmodels 0.11.1, and for AUC, utilizing Empirical Bootstrap with 1000 replicates. The ROC curves were drawn according to the sensitivity versus 1–specificity utilizing the packages of Scikit-learn 0.23.2 and Matplotlib 3.3.1. The deep learning provided a malignancy score ranging from 0 to 1 with a cutoff over 0.5 for classifying an eyelid tumor as being malignant.

Unweighted Cohen’s κ coefficients were calculated to compare the classification results of the system with the ground truth. The Kappa result is interpreted as follows: values ≤0 as indicating no agreement, 0.01–0.20 as slight, 0.21–0.40 as fair, 0.41– 0.60 as moderate, 0.61–0.80 as substantial, and 0.81–1.00 as almost perfect agreement^43^. The differences in sensitivities, specificities, and accuracies between the deep learning system and ophthalmologists were analyzed using the McNemar test. All statistical tests were 2-sided and the results were considered statistically significant at the level of *p* < 0.05. Statistical analyses were carried out using Python 3.7.8 (Wilmington, Delaware, USA).

### Reporting summary

Further information on research design is available in the Nature Research Reporting Summary linked to this article.

## Supplementary information

Supplementary Information

Reporting Summary

## Data Availability

The main data supporting the results of this study are available in the manuscript and its Supplementary Information. The raw datasets from the Ningbo Eye Hospital, Jiangdong Eye Hospital, and Zunyi First People’s Hospital cannot be made available due to hospital regulation restrictions and patient privacy concerns. Some anonymized data may be available for research purposes from the corresponding authors on reasonable request.

## Code availability

The code and example data used in this study can be accessed at GitHub (https://github.com/jiangjiewei/EyelidTumors-Source).
