# Supplementary material for: Artificial intelligence to detect malignant eyelid tumors from photographic images
Source: NPJ Digit Med. 2022 Mar 2;5:23. doi: 10.1038/s41746-022-00571-3 (PMC8891262; doi:10.1038/s41746-022-00571-3)
Supplement: Supplementary file 1 — Supplementary Information [file 41746_2022_571_MOESM1_ESM.pdf]

Title: Artificial intelligence to detect malignant eyelid tumors from photographic images

Supplementary Information

1. **Supplementary Figure 1 Confusion matrices of four deep learning algorithms in the internal and external test sets.** a, Internal test set. b, External test set. BET, benign eyelid tumor. MET, malignant eyelid tumor.

2. **Supplementary Figure 2. Visualization by t-SNE for presenting the separability of the features learned by four different deep learning algorithms.** a, Internal test set. b, External test set. t-SNE, t-distributed stochastic neighbor embedding. BET, benign eyelid tumor. MET, malignant eyelid tumor.

3. **Supplementary Figure 3. Performance of the deep learning system in images of eyelid tumors of uncertain malignant nature.** ETUMA, eyelid tumors of uncertain malignant nature.

4. **Supplementary Figure 4. False-negative and false-positive findings by the deep learning system in the internal and external test sets.** BCC, basal cell carcinomas. SCC, squamous cell carcinomas. SCP, squamous cell papilloma.

5. **Supplementary Figure 5. Examples of incorrectly classified images by the deep learning system.** a, Images of malignant eyelid tumors (MET) were misclassified by the system as benign eyelid tumors (BET). b, Images of BET were misclassified by the system as MET.

6. **Supplementary Figure 6. Confusion matrices of three ophthalmologists in the external test set.** BET, benign eyelid tumor. MET, malignant eyelid tumor.

7. **Supplementary Figure 7. Diagram showing an overview of the proposed eyelid tumor detection system.** Convolution layers are employed to filter an input image and extract effective features. Feature maps show the output of previous layers. Region proposals indicate a network that outputs interesting regions (eyelid tumors) from feature maps. ROI pooling is a layer that conducts maximum pooling in region proposals of non-uniform sizes for obtaining fixed-size feature maps. RCNN, region-based convolutional neural network. ROI, region of interest.

8. **Supplementary Figure 8. Flow diagram of the development and assessment of the deep learning classification system.**

9. **Supplementary Table 1 Performance of the deep learning system vs. ophthalmologists in the external test set.**

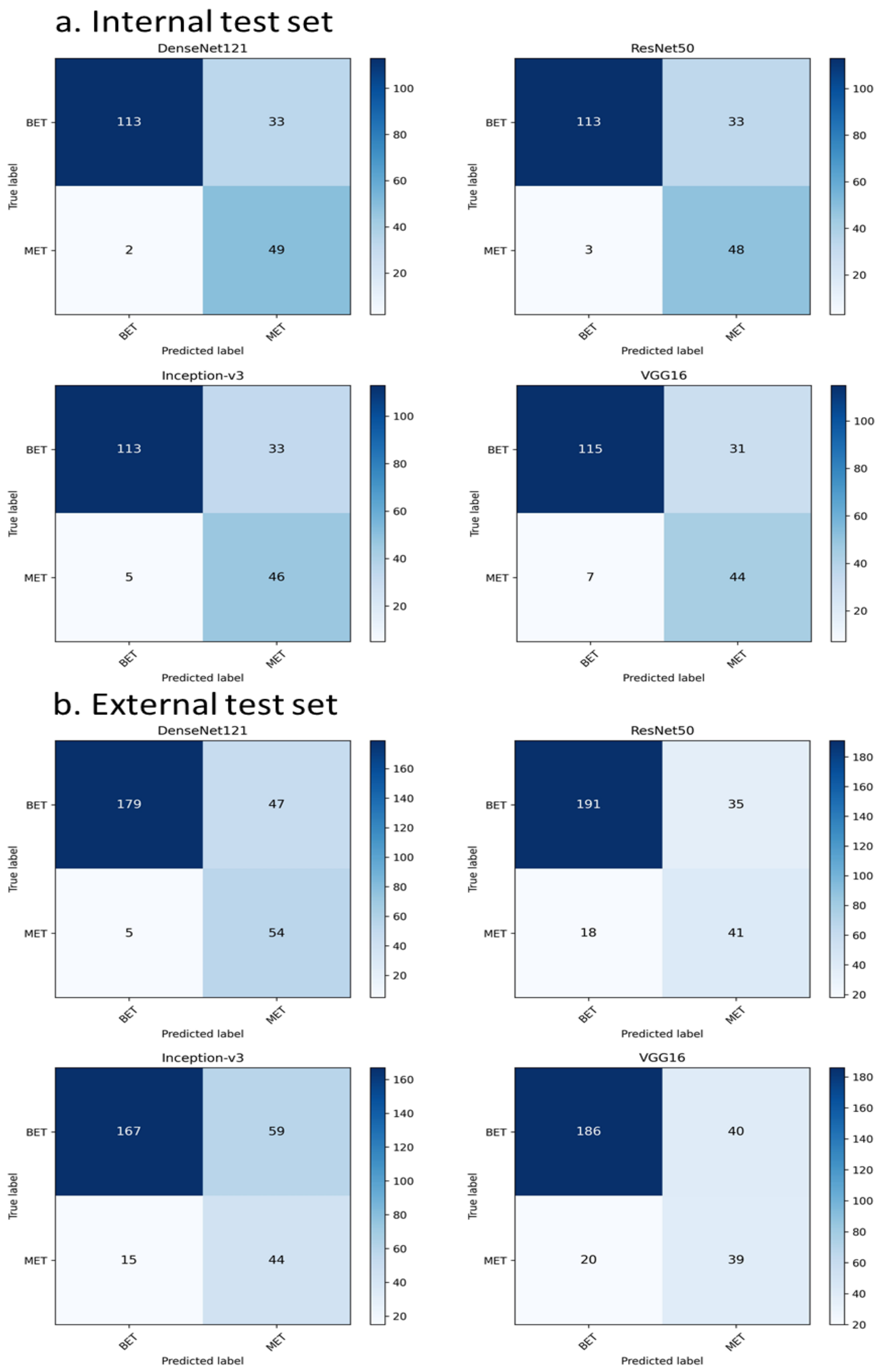

Supplementary Figure 1. Confusion matrices of four deep learning algorithms in the internal and external test sets. a, Internal test set. b, External test set. BET, benign eyelid tumor. MET, malignant eyelid tumor.

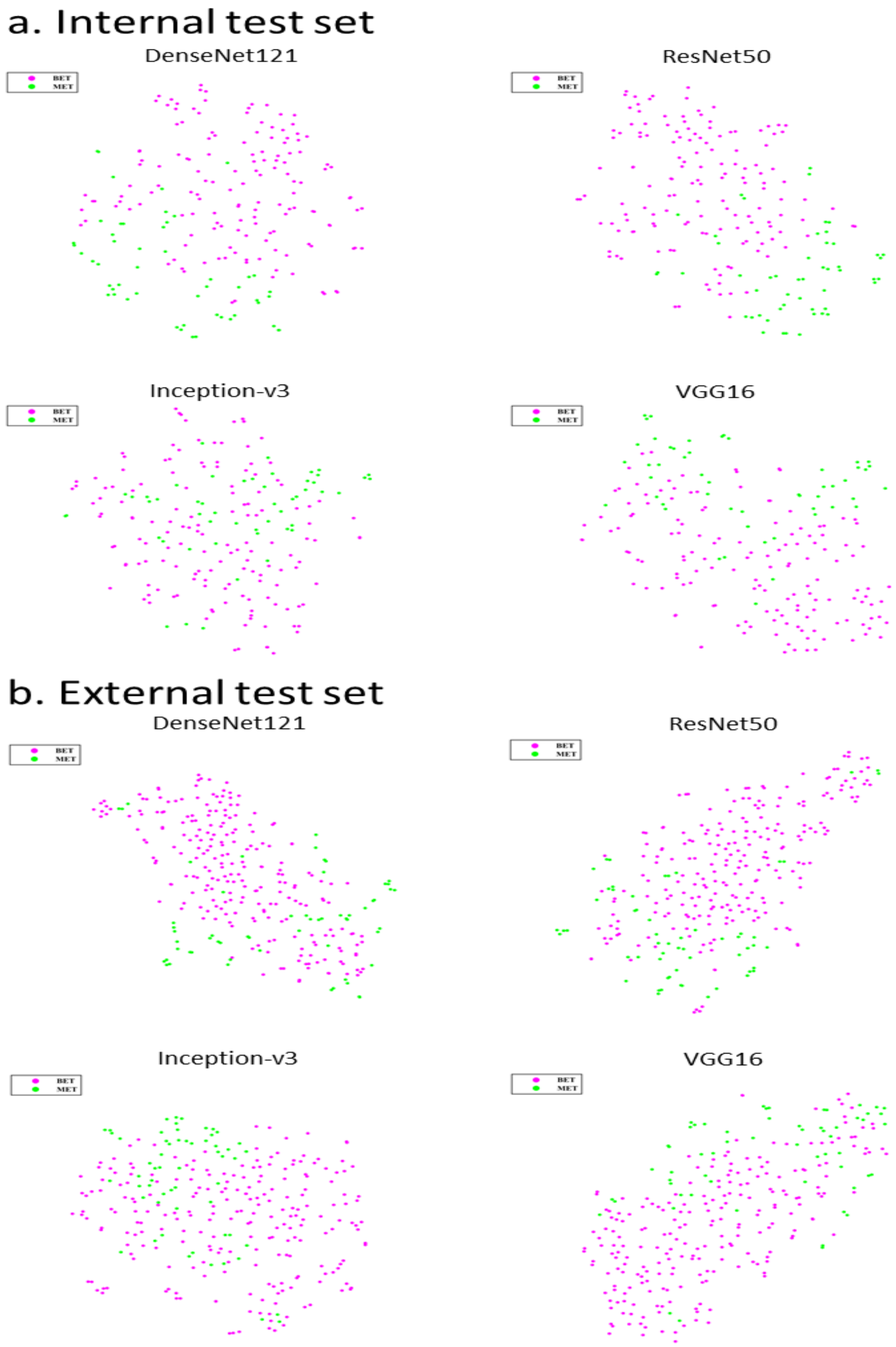

Supplementary Figure 2. Visualization by t-SNE for presenting the separability of the features learned by four different deep learning algorithms. a, Internal test set. b, External test set. t-SNE, t-distributed stochastic neighbor embedding. BET, benign eyelid tumor. MET, malignant eyelid tumor.

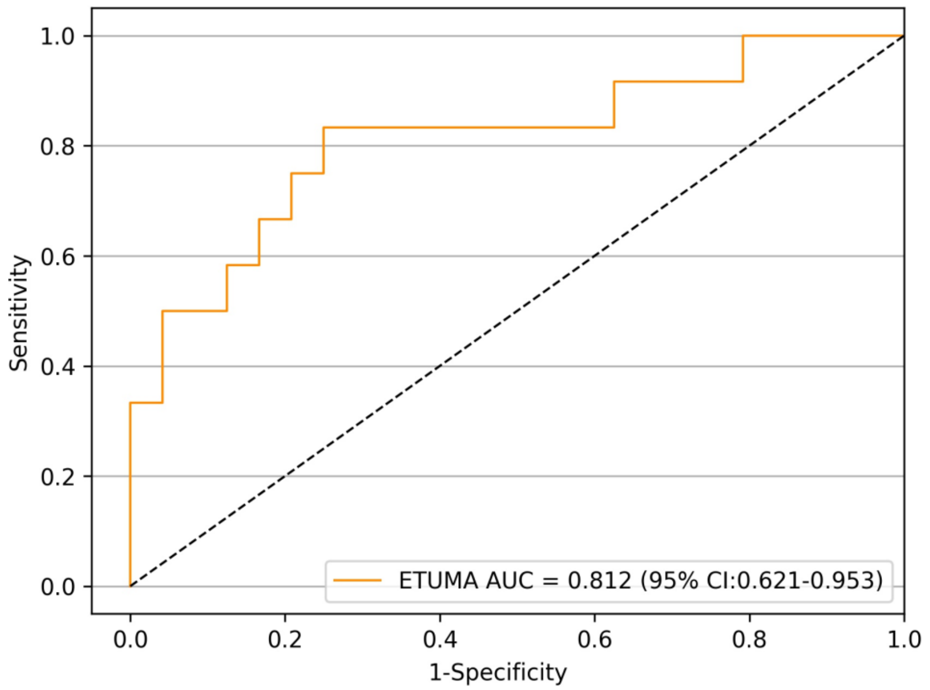

Supplementary Figure 3. Performance of the deep learning system in images of eyelid tumors of uncertain malignant nature. ETUMA, eyelid tumors of uncertain malignant nature.

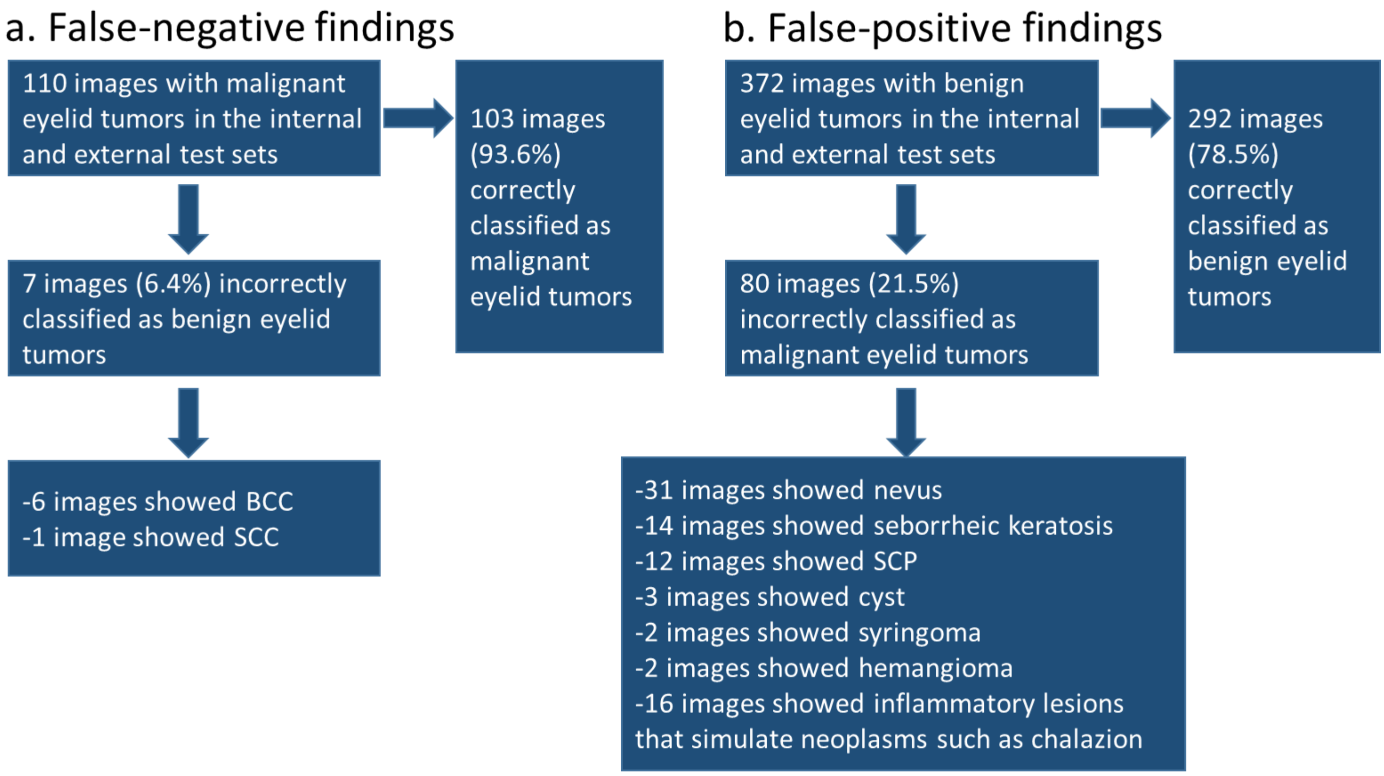

Supplementary Figure 4. False-negative and false-positive findings by the deep learning system in the internal and external test sets. BCC, basal cell carcinomas. SCC, squamous cell carcinomas. SCP, squamous cell papilloma.

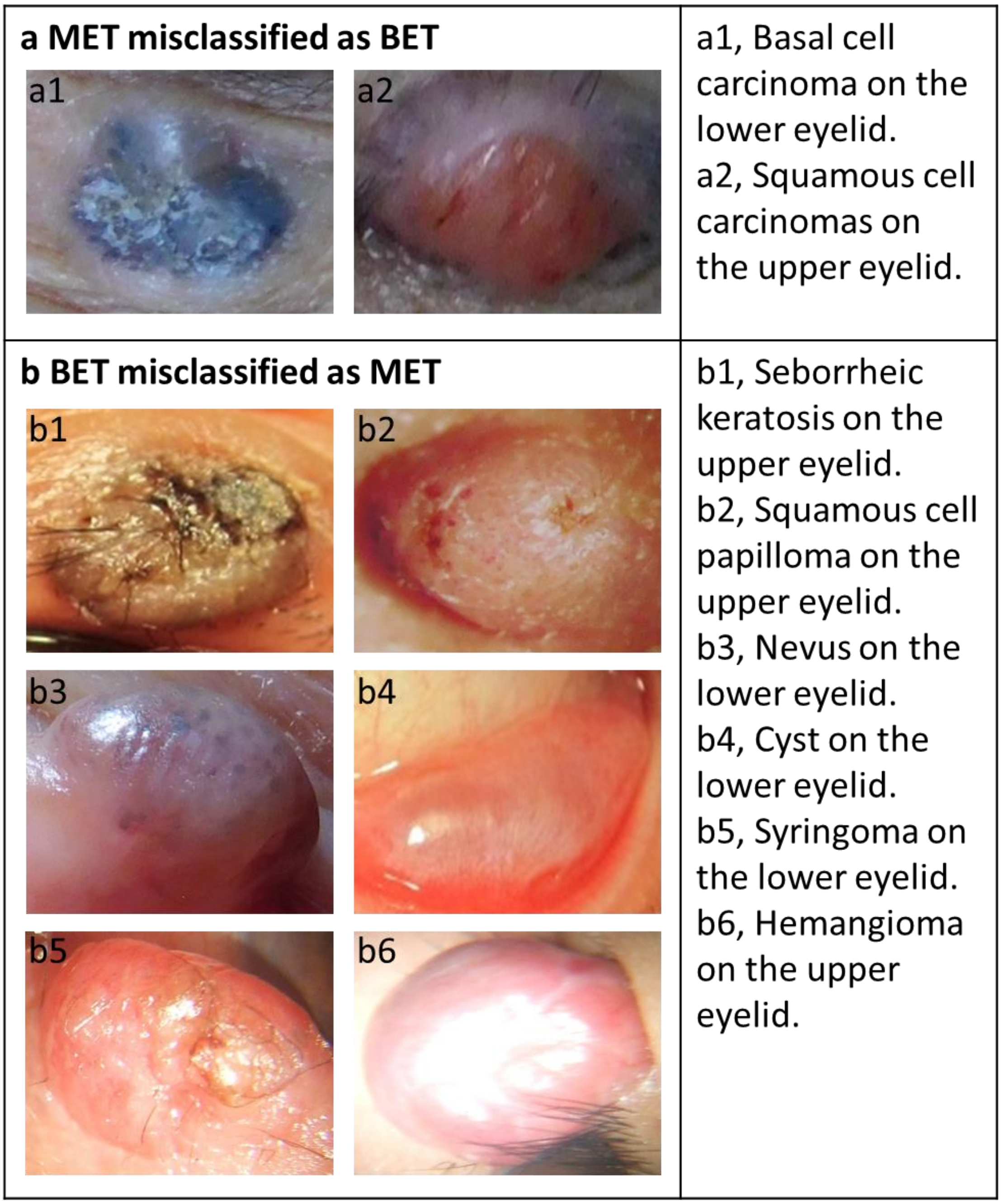

Supplementary Figure 5. Examples of incorrectly classified images by the deep learning system. a, Images of malignant eyelid tumors (MET) were misclassified by the system as benign eyelid tumors (BET). b, Images of BET were misclassified by the system as MET.

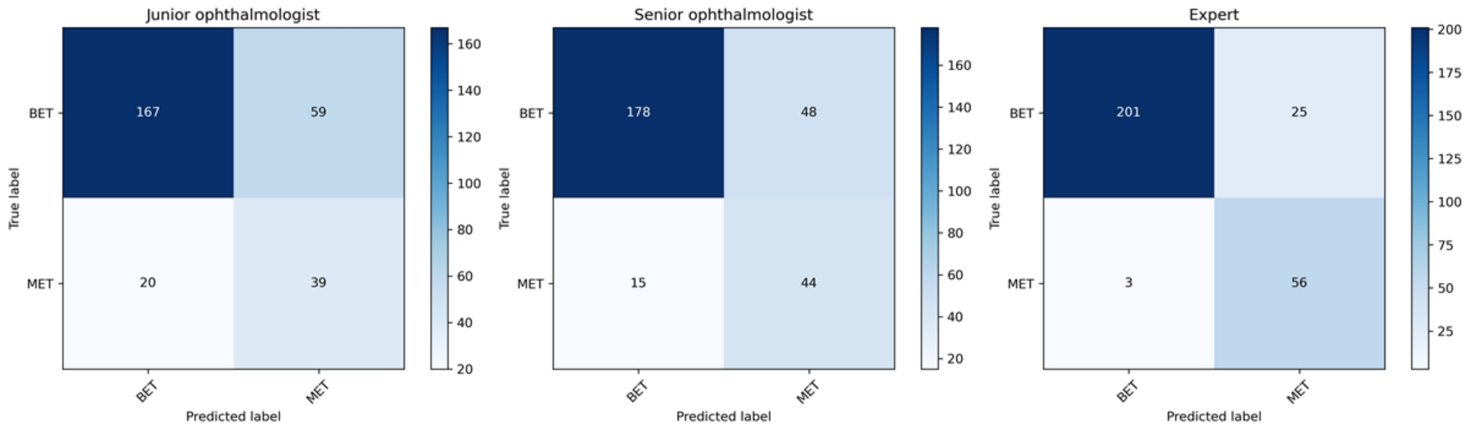

Supplementary Figure 6. Confusion matrices of three ophthalmologists in the external test set. BET, benign eyelid tumor. MET, malignant eyelid tumor.

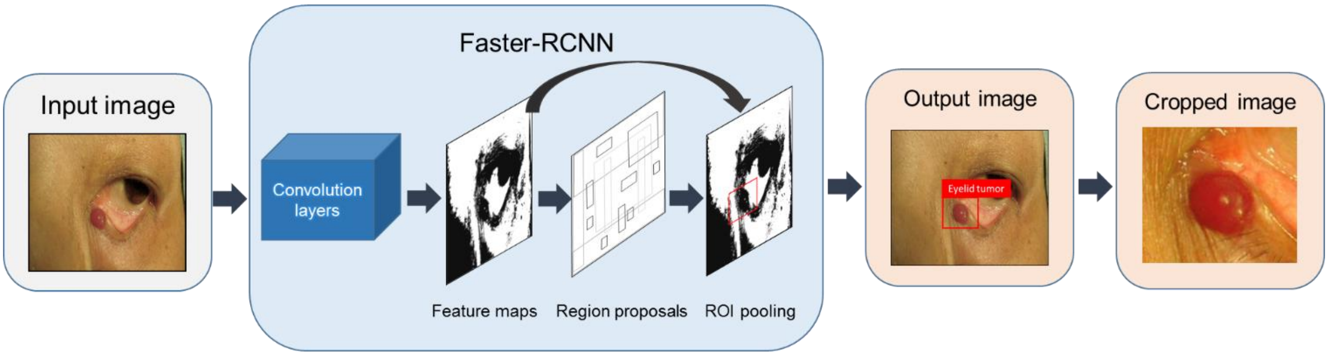

Supplementary Figure 7. Diagram showing an overview of the proposed eyelid tumor detection system. Convolution layers are employed to filter an input image and extract effective features. Feature maps show the output of previous layers. Region proposals indicate a network that outputs interesting regions (eyelid tumors) from feature maps. ROI pooling is a layer that conducts maximum pooling in region proposals of non-uniform sizes for obtaining fixed-size feature maps. RCNN, region-based convolutional neural network. ROI, region of interest.

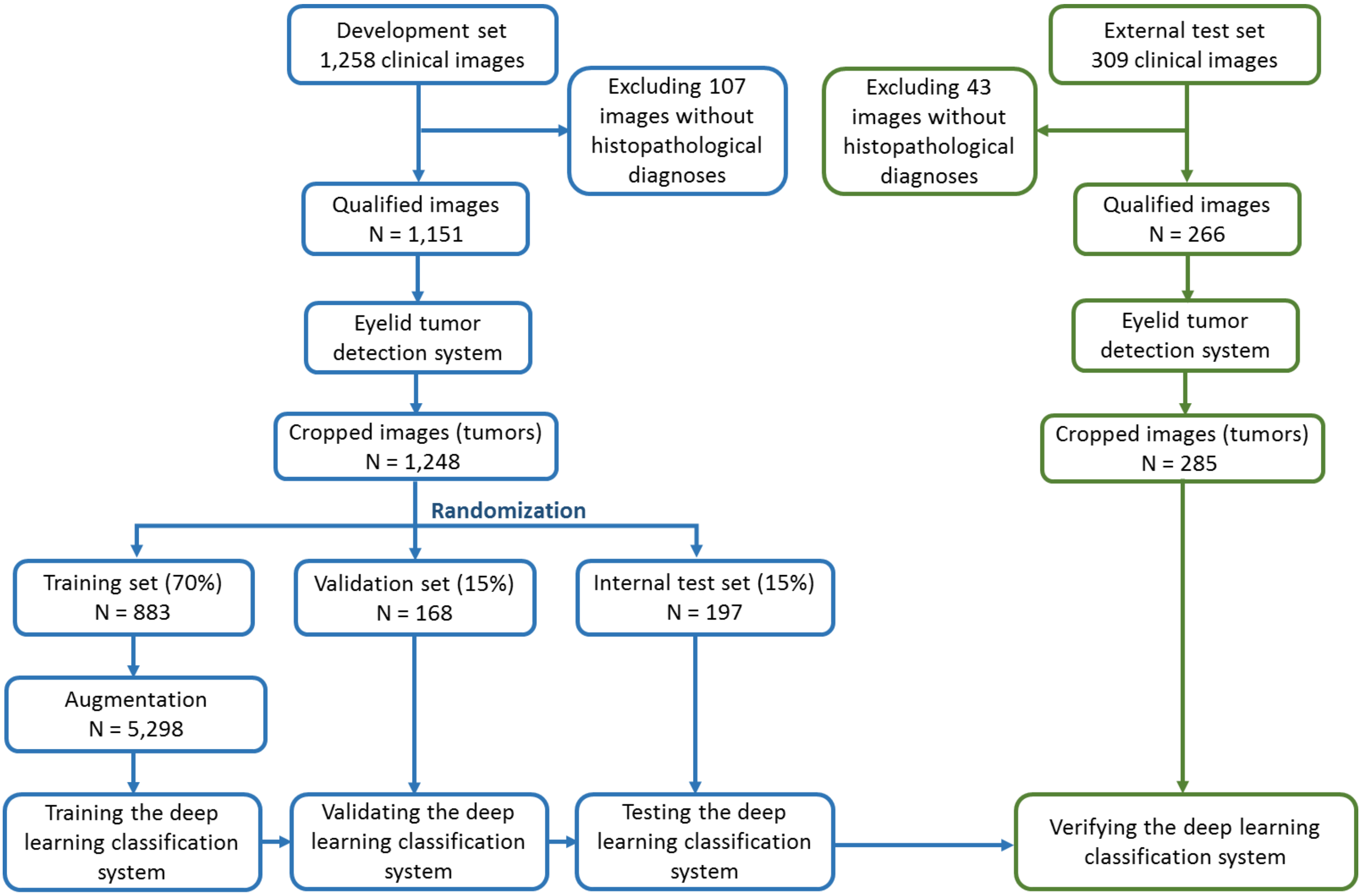

Supplementary Figure 8. Flow diagram of the development and assessment of the deep learning classification system.

Supplementary Table 1 Performance of the deep learning system vs. ophthalmologists in the external test set.

	Deep learning system	Junior	Senior	Expert	*P1*	*P2*	*P3*
Sensitivity (95% CI)	91.5% (84.4-98.6)	66.1% (54.0-78.2)	74.6% (63.5-85.7)	94.9% (89.3-100)	< 0.001	0.006	0.688
Specificity (95% CI)	79.2% (73.9-84.5)	73.9% (68.2-79.6)	78.8% (73.4-84.1)	88.9% (84.8-93.0)	0.195	1.000	0.004
Accuracy (95% CI)	81.8% (77.3-86.2)	72.3% (67.1-77.5)	77.9% (73.1-82.7)	90.2% (86.7-93.6)	0.006	0.228	0.003

"Junior" indicates an ophthalmologist with 3 years of clinical experience. "Senior" indicates an ophthalmologist with 7 years of clinical experience. "Expert" indicates an ophthalmologist with 15 years of clinical experience. *P1* denotes the p-value that was calculated between the system and junior ophthalmologist using the two-sided McNemar test. *P2* denotes the p-value that was calculated between the system and senior ophthalmologist using the two-sided McNemar test. *P3* denotes the p-value that was calculated between the system and the expert using the two-sided McNemar test. CI, confidence interval.
