# Supplementary material for: Artificial intelligence to detect malignant eyelid tumors from photographic images
Source: NPJ Digit Med. 2022 Mar 2;5:23. doi: 10.1038/s41746-022-00571-3 (PMC8891262; doi:10.1038/s41746-022-00571-3)
Supplement: Supplementary file 2 — Reporting Summary [file 41746_2022_571_MOESM2_ESM.pdf]

nature portfolio

Corresponding author(s): Zhongwen Li
Last updated by author(s): Jan 26, 2022

Reporting Summary

Nature Portfolio wishes to improve the reproducibility of the work that we publish. This form provides structure for consistency and transparency in reporting. For further information on Nature Portfolio policies, see our Editorial Policies and the Editorial Policy Checklist.

Statistics

For all statistical analyses, confirm that the following items are present in the figure legend, table legend, main text, or Methods section.

n/a	Confirmed	
☐	☒	The exact sample size (n) for each experimental group/condition, given as a discrete number and unit of measurement
☐	☒	A statement on whether measurements were taken from distinct samples or whether the same sample was measured repeatedly
☐	☒	The statistical test(s) used AND whether they are one- or two-sided<br>*Only common tests should be described solely by name; describe more complex techniques in the Methods section.*
☒	☐	A description of all covariates tested
☒	☐	A description of any assumptions or corrections, such as tests of normality and adjustment for multiple comparisons
☐	☒	A full description of the statistical parameters including central tendency (e.g. means) or other basic estimates (e.g. regression coefficient) AND variation (e.g. standard deviation) or associated estimates of uncertainty (e.g. confidence intervals)
☐	☒	For null hypothesis testing, the test statistic (e.g. F, t, r) with confidence intervals, effect sizes, degrees of freedom and P value noted<br>*Give P values as exact values whenever suitable.*
☒	☐	For Bayesian analysis, information on the choice of priors and Markov chain Monte Carlo settings
☒	☐	For hierarchical and complex designs, identification of the appropriate level for tests and full reporting of outcomes
☐	☒	Estimates of effect sizes (e.g. Cohen's d, Pearson's r), indicating how they were calculated

Our web collection on statistics for biologists contains articles on many of the points above.

Software and code

Policy information about availability of computer code

Data collection	No software was used for data collection.
Data analysis	The performance of the ETDS was evaluated by calculating the average precision (AP) score using mmdetection 2.10.0. The sensitivity, specificity, accuracy, and area under the receiver operating characteristic curve (AUC) were calculated to assess the performance of the deep learning classification system. The 95% confidence intervals (CIs) of sensitivity, specificity, and accuracy were estimated with the Wilson Score approach utilizing a package of Statsmodels 0.11.1, and for AUC, utilizing Empirical Bootstrap with 1000 replicates. The receiver operating characteristic (ROC) curves were drawn according to the sensitivity versus 1 – specificity utilizing the packages of Scikit-learn 0.23.2 and Matplotlib 3.3.1. Unweighted Cohen's κ coefficients were calculated to compare the classification results of the system with the ground truth. The differences in sensitivities, specificities, and accuracies between the deep learning system and ophthalmologists were analyzed using the McNemar test. All statistical tests were 2-sided and the results were considered statistically significant at the level of $p < 0.05$. Statistical analyses were carried out using Python 3.7.8 (Wilmington, Delaware, USA).

For manuscripts utilizing custom algorithms or software that are central to the research but not yet described in published literature, software must be made available to editors and reviewers. We strongly encourage code deposition in a community repository (e.g. GitHub). See the Nature Portfolio guidelines for submitting code & software for further information.

Data

Policy information about availability of data

All manuscripts must include a data availability statement. This statement should provide the following information, where applicable:

- Accession codes, unique identifiers, or web links for publicly available datasets
- A description of any restrictions on data availability
- For clinical datasets or third party data, please ensure that the statement adheres to our policy

The code and example data used in this study can be accessed at GitHub (https://github.com/jiangjiewei/EyelidTumors-Source).The main data supporting the results of this study are available in the manuscript and its Supplementary Information. The raw datasets from the Ningbo Eye Hospital, Jiangdong Eye Hospital, and Zunyi First People's Hospital cannot be made available due to hospital regulation restrictions and patient privacy concerns. Some anonymized data may be available for research purposes from the corresponding authors on reasonable request.

Field-specific reporting

Please select the one below that is the best fit for your research. If you are not sure, read the appropriate sections before making your selection.

☒ Life sciences ☐ Behavioural & social sciences ☐ Ecological, evolutionary & environmental sciences

For a reference copy of the document with all sections, see nature.com/documents/nr-reporting-summary-flat.pdf

Life sciences study design

All studies must disclose on these points even when the disclosure is negative.

Sample size	After excluding 150 photographic images without histopathological diagnoses, a total of 1,417 images with 1,533 eyelid tumors delineated by tight bounding boxes were used to establish and evaluate the ETDS. A total of 1,533 cropped images (1,161 images of benign tumors and 372 images of malignant tumors) created by the ETDS were leveraged to develop and assess the deep learning classification system.
Data exclusions	Images without histopathological diagnoses were excluded from the study.
Replication	The PyTorch deep learning framework (version 1.6.0) was used to train, validate, and test our models. The DenseNet121, ResNet50, Inception-v3, and VGG16 were trained using 4 Nvidia 2080TI graphics processing units. The mini-batch size was set at 32 on each GPU to gain 128 images in one iteration. The average value of these samples was computed to update the trainable parameters. A variation of the stochastic gradient descent algorithm, adaptive moment estimation (ADAM) optimizer, was used with an initial learning rate at 0.001, β1 of 0.9, β2 of 0.999, and a weight decay of 1e-4. Each algorithm was trained for 80 epochs. During the training process, accuracy and cross-entropy loss were calculated on the training and validation sets after each epoch and utilized as a reference for model selection. Each time the accuracy increased or cross-entropy loss decreased, a checkpoint saved the model state and corresponding weight matrix. The model with the highest validation accuracy was selected for use on the test sets.
Randomization	The cropped images created by the ETDS using the NEH dataset were randomly split at a 7:1.5:1.5 ratio for training, validation, and testing of a deep learning classification system. No overlap was allowed among training, validation, and internal test sets.
Blinding	To reflect the real level of the ophthalmologists in routine clinical practices, they were not informed that they competed with the system to avoid bias from the competition.

Reporting for specific materials, systems and methods

We require information from authors about some types of materials, experimental systems and methods used in many studies. Here, indicate whether each material, system or method listed is relevant to your study. If you are not sure if a list item applies to your research, read the appropriate section before selecting a response.

Materials & experimental systems

n/a	Involved in the study	
☒	☐	Antibodies
☒	☐	Eukaryotic cell lines
☒	☐	Palaeontology and archaeology
☒	☐	Animals and other organisms
☐	☒	Human research participants
☒	☐	Clinical data
☒	☐	Dual use research of concern

Methods

n/a	Involved in the study	
☒	☐	ChIP-seq
☒	☐	Flow cytometry
☒	☐	MRI-based neuroimaging

Human research participants

Policy information about studies involving human research participants

Population characteristics	For developing a deep learning system, a total of 1,258 photographic images (675 patients) were collected at Ningbo Eye Hospital (NEH). The NEH dataset included subjects who presented for eye examinations and ophthalmology consultations due to the discovery of eyelid tumors. The images were captured between January 2010 and March 2021 using ordinary digital cameras. To better confirm the effectiveness and generalizability of the deep learning system, an additional dataset including 248 photographic images (129 patients) collected at Jiangdong Eye Hospital (JEH) and 61 photographic images (47 patients) collected at Zunyi First People's Hospital (ZFPH) were used to externally assess the system.
Recruitment	A total of 1,417 images collected from NEH, JEH, and ZFPH were used to establish and evaluate the ETDS. A total of 1,533 cropped images (1,161 images of benign tumors and 372 images of malignant tumors) created by the ETDS were leveraged to develop and assess the deep learning classification system.
Ethics oversight	Approval from the institutional review board of NEH was obtained (identifier, 2021-qtky-44), and the study protocol was performed following the Declaration of Helsinki principles. Informed consent was waived by the ethics committee of NEH due to the retrospective nature of the data collection and the use of de-identified images.

Note that full information on the approval of the study protocol must also be provided in the manuscript.
